# A Paper-Based Sandwich Format Hybridization Assay for Unlabeled Nucleic Acid Detection Using Upconversion Nanoparticles as Energy Donors in Luminescence Resonance Energy Transfer

**DOI:** 10.3390/nano5041556

**Published:** 2015-09-24

**Authors:** Feng Zhou, M. Omair Noor, Ulrich J. Krull

**Affiliations:** Chemical Sensors Group, Department of Chemical and Physical Sciences, University of Toronto Mississauga, 3359 Mississauga Road, Mississauga, ON L5L 1C6, Canada; E-Mails: forrest.zhou@utoronto.ca (F.Z.); omair.noor@utoronto.ca (M.O.N.)

**Keywords:** luminescence resonance energy transfer, upconversion nanoparticle, nucleic acid hybridization, sandwich assay, paper-based assay

## Abstract

Bioassays based on cellulose paper substrates are gaining increasing popularity for the development of field portable and low-cost diagnostic applications. Herein, we report a paper-based nucleic acid hybridization assay using immobilized upconversion nanoparticles (UCNPs) as donors in luminescence resonance energy transfer (LRET). UCNPs with intense green emission served as donors with Cy3 dye as the acceptor. The avidin functionalized UCNPs were immobilized on cellulose paper and subsequently bioconjugated to biotinylated oligonucleotide probes. Introduction of unlabeled oligonucleotide targets resulted in a formation of probe-target duplexes. A subsequent hybridization of Cy3 labeled reporter with the remaining single stranded portion of target brought the Cy3 dye in close proximity to the UCNPs to trigger a LRET-sensitized emission from the acceptor dye. The hybridization assays provided a limit of detection (LOD) of 146.0 fmol and exhibited selectivity for one base pair mismatch discrimination. The assay was functional even in undiluted serum samples. This work embodies important progress in developing DNA hybridization assays on paper. Detection of unlabeled targets is achieved using UCNPs as LRET donors, with minimization of background signal from paper substrates owing to the implementation of low energy near-infrared (NIR) excitation.

## 1. Introduction

Upconversion nanoparticles (UCNPs) are a category of luminescent materials with intriguing properties for development of bioanalytical assays [1]. Such attributes include physical and chemical stability, tunable emission profile over the UV-vis range, and excitation by near-infrared radiation (typically 980 nm), which greatly reduces scattering and background autofluorescence [2]. Upconversion nanoparticles can participate in non-radiative energy transfer between a donor and an acceptor in a process that is similar to fluorescence resonance energy transfer (FRET) [3]. Since the emission of UCNPs is formally described as luminescence, the resonance energy transfer phenomenon using UCNPs as energy donors is referred to as luminescence resonance energy transfer (LRET) [4,5]. The determination of a resonance energy transfer signal is typically based on a ratiometric calculation that compares the acceptor and donor emission, and this approach tends to provide superior precision when considering bioassay development [6,7].

The first LRET-based bioassay was reported in 2005, where a streptavidin detection assay was developed using biotinylated UCNPs as energy donor and gold nanoparticles as luminescence quencher [8]. Various LRET-based detection strategies for different targets have been reported. For example, Hu *et al.* developed a device for trinitrotoluene (TNT) detection using the quenching effect of TNT on polyethylenimine-coated UCNPs that were immobilized as arrays on silicon wafers [9]. Graphene oxide has also been used as a quencher in LRET-based DNA hybridization assays [10]. Ding *et al.* developed a Fe^3+^ sensor by using the LRET signal between UCNPs and a Rhodamine B derivative, which was based on a substantial change in absorbance spectrum that was induced by chelation with Fe^3+^ [11]. Recently, Doughan *et al.* reported a LRET-based DNA hybridization assay on paper using QD-labeled DNA as energy acceptors to improve the LRET efficiency [12]. Liu *et al.* developed a LRET-based DNA hybridization assay in solution by using UCNPs that were conjugated with probe DNA as energy donors and dye-labeled DNA as energy acceptors [13].

Paper-based assays have attracted attention for practical cost-effective applications, such as desired for point-of-care and consumer diagnostics [14,15]. Paper offers three-dimensional fibrous structures of large surface area, a capillary wicking effect for fluid transport, easy chemical modification, and potential for patterning, particularly when combined with printing of hydrophobic barriers [7,16,17]. A recent report by Noor *et al.* suggested that a further advantage for paper was associated with FRET signal amplification. They developed a paper-based analytical device (PADs) for a ratiometric fluorescence transduction of amplicons that were generated using thermophilic helicase-dependent amplification (tHDA) method using immobilized quantum dots as donors that were FRET-paired with Cy3 fluorophore. The Cy3 fluorophore was associated with a reporter strand in a sandwich-based nucleic acid hybridization assay format. Zeptomole quantities of target oligonucleotide strands were detectable using a readout platform that comprised handheld excitation source and an iPad camera [7,18]. Connelly *et al.* developed a hand-held paper-based device, which integrated sample preparation and loop-mediated isothermal amplification (LAMP). This prototype minimized pipetting steps and the detection signal made use of UV light as an excitation source and a limit of detection of five *E. coli* cells in whole blood sample was reached [19].

In previous work investigating LRET, we demonstrated a DNA hybridization assay on paper using dye-labeled oligonucleotides as targets [17]. Superior sensitivity and detection limit were achieved in comparison to an analogous DNA hybridization assay using quantum dots (QDs) as energy donors due to reduction of background signal by use of 980 nm laser excitation. We further extended this method to a two-plex DNA hybridization assay using two different dye-labeled targets that interacted with two different wavelengths of emission from one form of UCNP [16]. In all of these experiments, the oligonucleotide targets were covalently conjugated with fluorescent dye as the energy acceptor. Labeling of target DNA with molecular dyes in a real samples of complex composition can be challenging, and adds time and cost to analysis. One solution to this issue is implementation of sandwich assays where a reporter oligonucleotide associates with a probe-target hybrid [20]. There are many reports of such DNA hybridization assays for solution phase work, and some for FRET using QDs. Very recently, Doughan *et al.* published the first report of a covalently immobilized LRET-based DNA hybridization sandwich assay on paper using QD-labeled DNA as a reporter to avoid labeling of the target DNA [12]. It was noted that the kinetics of hybridization with the reporter were slowed by the presence of the relatively massive QD labels, and there remain concerns about the cost and toxicity of QDs as a label for widespread applications in the consumer market [21,22].

Herein, for the first time, we report the development of a LRET-based sandwich format DNA hybridization assay that makes use of conventional molecular dyes as acceptors that are associated with the reporter strands. The UCNPs were functionalized and immobilized onto paper according to our previously published method [16,17]. Biotinylated probe oligonucleotide for the *E. coli* hylA sequence was subsequently immobilized onto avidin coated UCNPs. After hybridization of the unlabeled target DNA with probe, dye-labeled reporter was hybridized with the remaining single stranded portion of the target DNA. The hybridization enabled the proximity between the UCNP and dye that was required for LRET, and excitation of the UCNPs was stimulated by radiation from a 980 nm diode laser. The LRET signal was proportional to the quantity of target DNA. The ratiometric method allowed for quantification of the un-labeled target oligonucleotide with a competitive limit of detection (LOD), and selectivity for one base pair mismatch (1BPM) target discrimination, using samples containing various interferents, and even samples prepared using undiluted serum.

## 2. Results and Discussion

**Scheme 1:**
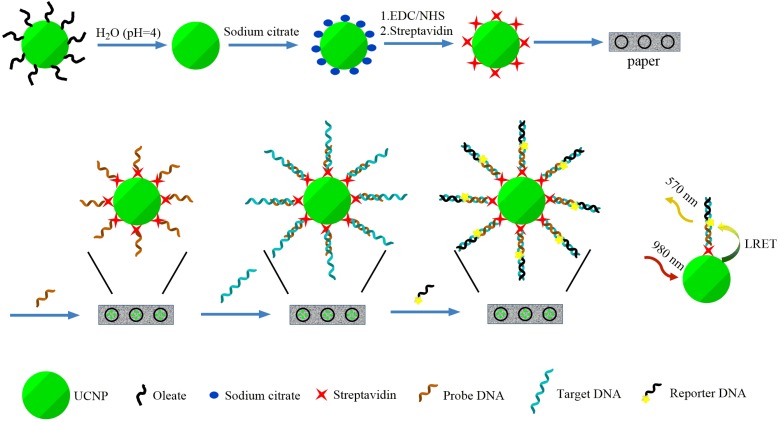
Sandwich format DNA assay on paper using upconversion nanoparticles (UCNPs) as energy donors in luminescence resonance energy transfer (LRET). The representation of molecules and nanoparticles is not to scale, and does not reflect the actual density of protein and probe sites.

The general format of the assays is shown in Scheme 1.

### 2.1. Synthesis and Functionalization of Core/Shell UCNPs

**Figure 1:**
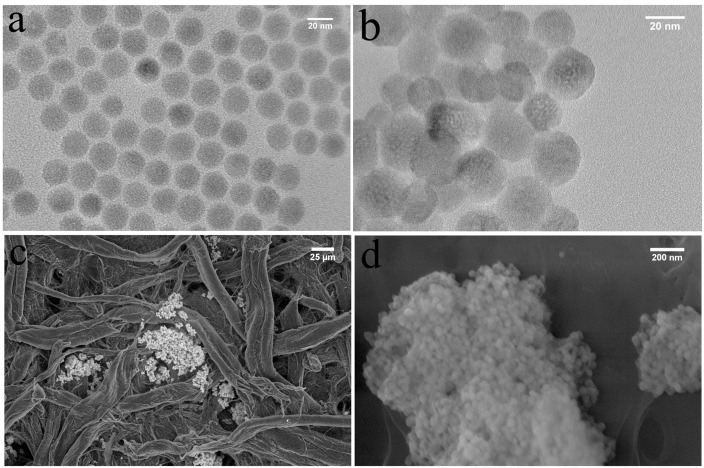
The characterization of oleate-coated UCNPs (OA-UCNPs) (**a**); avidin coated UCNP (**b**) in solution by TEM; Low magnification image of avidin coated UCNP on cellulose paper (**c**) and the zoomed in image (**d**).

UCNPs with green emission were synthesized using a well-established solvothermal method [23]. The as-synthesized oleate-coated UCNPs (OA-UCNPs) were well dispersed in hexane, and had an average diameter of 23.9 ± 3.6 calculated by measuring 100 randomly selected UCNPs nm (Figure 1a), and the hydrodynamic diameter given by Zetasizer is 25.0 ± 7.0 nm for 100% UCNPs. The hydrophobic OA-UCNPs required conversion to a water-soluble form before any biologically relevant investigations were possible. The OA-UCNPs were transformed to become water-soluble by first immersing in water with pH set to 4. After coating with sodium citrate, the UCNP surface was functionalized with carboxylic groups. Finally, the carboxyl groups of the citrate coated UCNPs were conjugated with avidin using EDC/NHS coupling. The fluorescent emission from Cy3 using direct excitation of the dye after conjugation with Cy3-labeled DNA was indicative of the successful conjugation of avidin. The avidin coated UCNPs can be dispersed in buffer and the morphology of the UCNPs after functionalization were generally unchanged with an average diameter of 25.3 ± 6.8 nm (Figure 1b). After adsorption onto paper and thoroughly washing in buffer, the UCNPs layer still adhered firmly on the cellulose fiber (Figure 1c,d).

**Figure 2:**
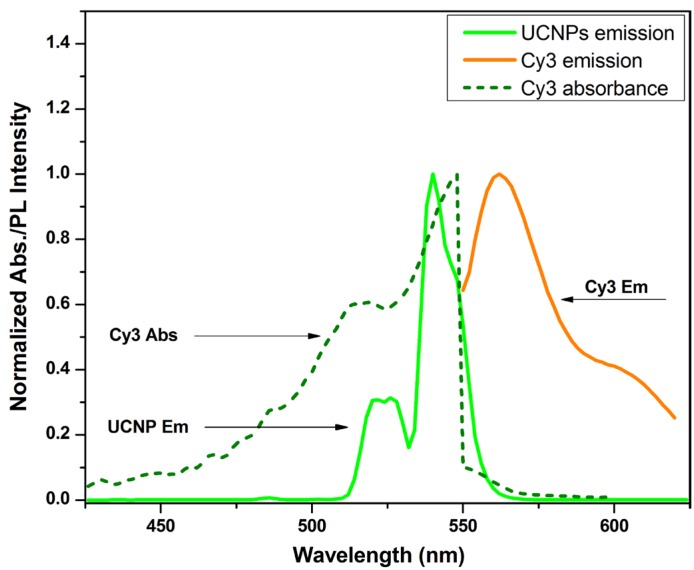
Normalized absorption (dashed lines) and emission spectra (solid lines) for the UCNP-Cy3 LRET pairs. Dashed grey line: Cy3 absorbance spectrum, absorbance peak: 548 nm; solid grey line: Cy3 emission spectrum, emission peak: 562 nm; solid dark line: UCNP emission spectrum, emission peak: 540 nm. The green area indicates the spectrum overlap between Cy3 absorbance and UCNP emission, and the yellow region indicates the Cy3 emission spectrum.

The normalized emission spectrum of UCNPs, Cy3, and the absorption spectrum of Cy3 are shown in Figure 2. The overlap of the green bands from UCNP and Cy3 emission and absorption envelopes indicate opportunity for LRET (Figure 2).

### 2.2. Performance of the Hybridization Assay

A ratiometric method based on the emission intensities of both UCNP (donor) and dye-labeled reporter DNA (acceptor) was used to quantify the amount of un-labeled target DNA. Ratiometric methods are less susceptible to variations caused by unpredictable factors, such as sample preparation and instrument responses, resulting in the improvement of precision and detection limit [7,24]. This approach has previously proven effective for DNA hybridization assays based on QD-FRET and UCNP-LRET systems on paper substrates [16,24]. The previous UCNP-LRET based DNA assays made use of target oligonucleotide that was pre-labeled with fluorescent dyes, which posed a significant drawback when considering the practicality of sample preparation [16,17]. To address this problem, a sandwich format hybridization assay has been implemented. A short oligonucleotide probe was designed to hybridize with a portion of the un-labeled target DNA, and a Cy3-labeled reporter oligonucleotide was prepared to hybridize with the remainder of the target DNA. Upon hybridization of the Cy3-reporter, the distance between UCNP and Cy3 provided the proximity for LRET. The amount of hybridized target DNA was determined from the ratiometric LRET signal intensity, which was proportional to the amount of hybridized target DNA.

It has been previously reported that the process of oligonucleotide hybridization on paper can be completed within 2 min [17,24]. The fast hybridization on paper can be attributed to the capillary wicking effect and the small pore sizes relative to diffusion constants of the oligonucleotides within the paper [24]. To ensure complete hybridization, 5 min was used for the sandwich format DNA hybridization assays.

**Figure 3:**
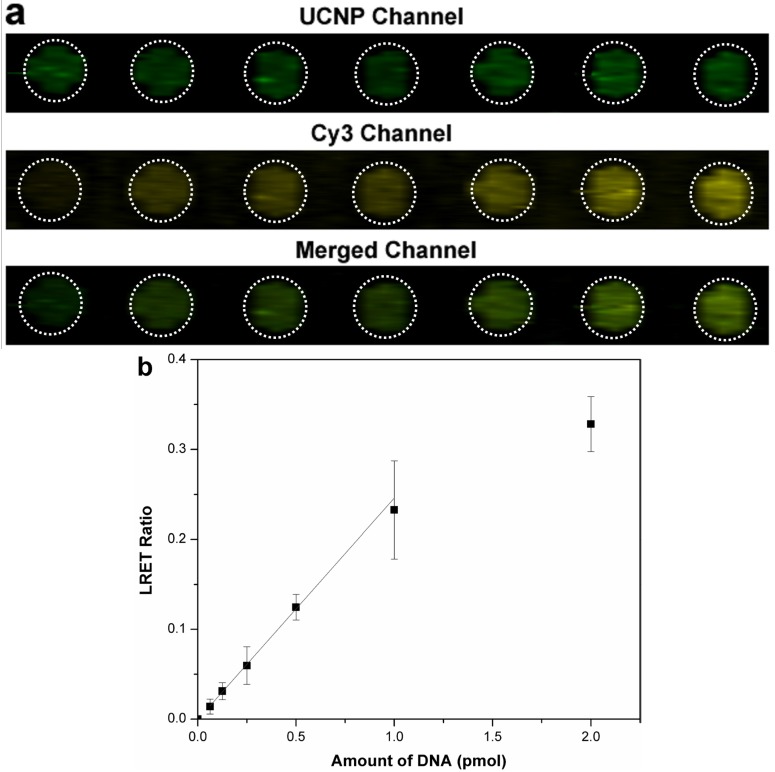
Quantification of un-labeled target DNA by LRET-sensitized sandwich assay using UCNPs to excite Cy3-labeled reporter DNA. (**a**) Pseudocolor fluorescence images collected via the filter for UCNP channel (520 ± 40 nm), the Cy3 filter channel (585 ± 40 nm), and the composite of the UCNP and Cy3 imaging channels corresponding to concentrations up to 2.00 (pmol) of the un-labeled target; (**b**) The calibration curve corresponding to the LRET responses was constructed using ImageJ to measure the emission intensity of the two channels (*n* = 4).

LRET intensities correlated with increasing concentrations of un-labeled target DNA as shown in Figure 3. The LRET ratio increased linearly up to 1.00 pmol, and the sensitivity of response attenuated when the amount of added target was greater than 1.00 pmol. A LOD of 146 fmol was calculated based on a signal that was 3 standard deviations higher than the background LRET ratio.

The LOD observed from this sandwich format hybridization assay was not as good as that reported for the analogous assay that implemented dye-labeled target DNA (LOD of 34 fmol) [17]. This reduction in performance is attributed to the lower LRET efficiency between UCNP and dye. There was an increased distance between the dye on the reporter DNA and UCNP relative to the dye-labeled target DNA, where the dye was at the proximal end of the DNA target. The four-fold higher LOD observed for the case of the sandwich assay format in comparison with the direct hybridization assay format is putatively attributed to the increase in the separation distance (*ca.* 6 nm assuming that oligonucleotide strands are placed orthogonal to the surface of UCNP) between the UCNP surface and the location of the acceptor dye (acceptor dye at the distal end), which is inherent in the sandwich assay configuration. A 6 nm increase in the donor-acceptor separation distance is expected to lower the efficiency of energy transfer given the strong dependency of energy transfer efficiency on the donor-acceptor separation distance. It is important to note that for the solid-phase assays, there is no definitive model that correlates the changes in the energy transfer efficiency with the donor-acceptor separation distance.

### 2.3. 1BPM Discrimination Assay

**Figure 4:**
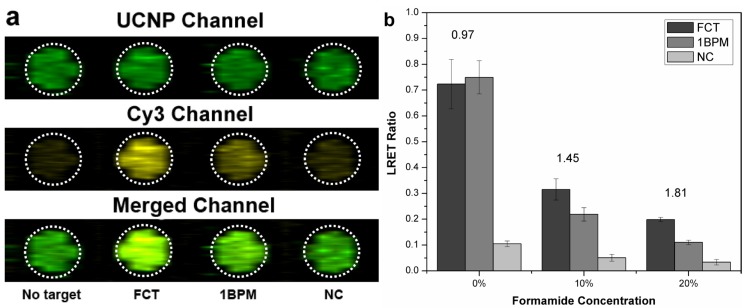
Evaluation of selectivity of the sandwich format hybridization assay for FCT and 1BPM targets using various concentrations of formamide, (**a**) Pseudocolor fluorescence images of the paper after treatment with buffer containing 20% (*v*/*v*) formamide for 10 min for assays of 4.0 μM NC, 1 BPM and FCT targets and 8.0 μM of the Cy3-labeled reporter DNA. The dashed circle indicates the region of interest measured using ImageJ; (**b**) LRET ratios based on the fluorescence intensity of images. The values above the columns indicate the signal ratio between FC and 1BPM targets (*n* = 4).

It is desirable that hybridization assays effectively discriminate between similar sequences of target oligonucleotides, and the most rigorous challenge is to achieve discrimination of a one base pair mismatched target. Control of solution ionic strength and addition of chaotropic agents can significantly improve the selectivity of hybridization. Lowering the concentration of salt destabilizes the hybridized DNA duplex, and mismatched hybrids have lower stability and can be eliminated [25]. Addition of formamide provides for a competitive hydrogen bond disruption, which helps to lower the melting temperature of the DNA duplex. The latter approach has been effective for the discrimination 1BPM dye-labeled targets without use of external heating. As can be seen in Figure 4, no significant differences in LRET signal were apparent for the sandwich assay after hybridization of FCT and 1BPM targets and the Cy3-labeled reporter in borate buffer. However, the signal intensity difference between the FCT and 1BPM targets became increasingly pronounced following treatment of the paper with increasing concentrations of formamide up to 20%. A maximum signal ratio of 1.81 was achieved after treatment of the paper with buffer containing 20% formamide. Background signal from the non-specific adsorption of NC was remained at a low level, indicating the minimized non-specific adsorption of targets. All experiments were done at room temperature (22 °C).

### 2.4. Hybridization Assay for Samples with a Complex Matrix Composition

**Figure 5:**
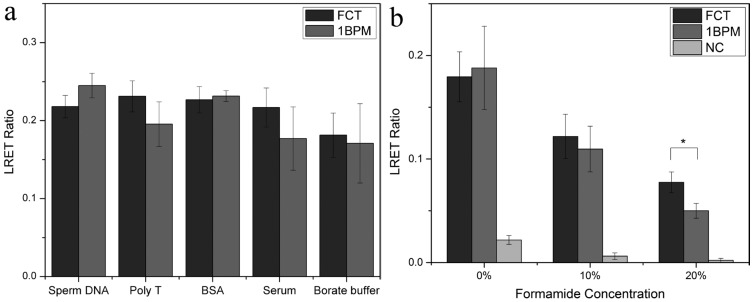
Evaluation of hybridization selectivity by detection of FCT and 1BPM target spiked in solutions containing different types of interferents; ~3.1 × 10^−6^ mol/L sheared salmon sperm DNA, 3.5 × 10^−6^ mol/L Poly T (T30) solution, 4.0 × 10^−4^ mol/L BSA, and undiluted goat serum. The final concentration of the spiked FCT target in the solutions was maintained at 0.5 × 10^−6^ mol/L (**a**); Evaluation of selectivity of the hybridization assay for FCT and 1BPM targets spiked in undiluted serum using various concentrations of formamide (**b**) (*n* = 4).

Hybridization assays often have reduced analytical performance when applied to samples that include a complicated matrix. FCT and 1BPM target were spiked into solutions containing several types of potential interferents, respectively, including: sheared salmon sperm DNA, Poly T (T30) solution, bovine serum albumin (BSA), and undiluted goat serum. The results shown in Figure 5a demonstrated that the ratiometric LRET signal of FCT and 1BPM target from all the sample solutions were the same within the precision of the experiments. This is consistent with the amelioration of non-specific adsorption by avidin coated UCNPs. Moreover, we further evaluated the hybridization stringency under the most challenging condition by spiking FCT and 1BPM target of the same concentration into undiluted serum respectively. It can be clearly seen that the signal from FCT and 1BPM group were similar after hybridization and treatment with 10% (*v*/*v*) formamide treatment (Figure 5b); However, significant difference can be observed with a maximum signal ratio of 1.55 between FCT and 1BPM can be reached after treating with 20% formamide for 10 min, indicating that even in matrix of complex composition, the 1BPM target can still be differentiated. Previously, Corstjens *et al.* reported a sandwich format DNA hybridization assay on lateral flow on nitrocellulose, where the LOD could reach up to 0.1 fmol and the assay time can be controlled within 20 min [26]. The LOD they achieved was better than our current results, and the total assay time was similar. In comparison, a ratiometric approach was used in our work instead of merely measuring the emission intensity of UCNP. The ratiometric method is believed to be less susceptible to variations caused by environment and instrument. Moreover, in our work, we not only evaluated the performance in detecting samples containing complex matrix, but also investigated hybridization stringency using samples containing single mismatched base.

## 3. Experimental Section

### 3.1. Reagents

Unless otherwise specified, chemicals and biological reagents were from Sigma-Aldrich. Cellulose chromatography paper (Whatman^®^ Grade 1, thickness: 0.18 mm) was used as substrate for immobilization of UCNPs. 50 mM borate buffer (BB, pH 9.25) containing 100 mM NaCl was used to prepare sample solutions. All buffer solutions were prepared using deionized water (Milli-Q, 18 MΩ·cm^−1^) and autoclaved prior to use. All oligonucleotide sequences were from Integrated DNA Technologies and purified using standard desalting or HPLC by the manufacturer (Coralville, IA, USA). The oligonucleotide sequences were dissolved in sterile Milli-Q water and stored at −20 °C.

The oligonucelotide sequences used for hybridization assays are listed in Table 1.

**Table 1:** Oligonucleotide sequences used in the hybridization assays ^a^.

*E. coli* hlyA probe	biotin-5′-TTC AGT TAA TCC TAC AAC-3′
*E. coli* hylA FCT	5′-GGT GCA GCA GAA AAA GTT GTA GGA TTA ACT GAA-3′
*E. coli* hylA 1BPM	5′-GGT GCA GCA GAA AAA GTT GTA G**A**A TTA ACT GAA-3′
*E. coli* hylA NC	5′-ATT TTG TCT GAA ACC CTG TAA GGA AAA TAA AGG-3′
*E. coli* hylA reporter	Cy3-5′-TTT TTC TGC TGC ACC-3′
Poly T (T30)	3′-TTTTTTTTTTTTTTTTTTTTTTTTTTTTTT-5′

^a^ FCT = fully complementary target, 1BPM = 1 base pair mismatched target, NC = non-complementary target. The mismatched base in the 1 BPM sequence is bolded and underlined.

### 3.2. Instrumentation and Characterization

The morphology of UCNPs was characterized using a high-resolution transmission electron microscope (HRTEM, JEOL 2010). The absorption spectra of Cy3 were collected using a HP8452A Diode-Array Spectrophotometer (Hewlett Packard Corporation, Palo Alto, CA, USA). The emission spectra of UCNPs and Cy3 were collected using a Quanta Master Photon Technology International spectrofluorimeter (London, ON, Canada) coupled with a 980 nm diode laser (800 mW, Laser Top Sale™, Beijing, China) and a xenon arc lamp for excitation (Ushio Incorporation, Cypress, CA, USA). Photoluminescence (PL) from paper was collected using a Nikon Eclipse L150 epifluorescence microscope (Nikon, Mississauga, ON, Canada) with a 40× Nikon WD Plan Fluor objective lens (NA 0.60). The microscope was custom modified with a 980 nm laser of the same model as was used with the spectrofluorimeter. Epifluorescence images from the emission of the UCNPs and organic dyes were collected using a H5784-20 PMT (Hamamatsu Corporation, Bridgewater, NJ, USA) as the detector by scanning the paper. D520/40 nm and D585/40 nm emission filters (Chroma Technologies Corp., Bellows Falls, VT, USA) were used to collect the green emission of UCNPs and the Cy3 LRET channels, respectively.

### 3.3. Procedures

Synthesis of β-NaYF_4_:2%Er^3+^, 18%Yb^3+^ core/shell UCNPs. β-NaYF_4_:2% Er^3+^, 18%Yb^3+^ core/shell UCNPs were synthesized using a reported procedure with minor modifications [27]. Briefly, Y(CH_3_CO_2_)_3_ hydrate (0.54 g, 1.6 mmol), Yb(CH_3_CO_2_)_3_ hydrate (0.16 g, 0.36 mmol) and Er(CH_3_CO_2_)_3_ hydrate (0.018 g, 0.040 mmol) were dissolved in a 100 mL three-neck round-bottom flask containing 30 mL octadecene and 12 mL oleic acid. The solution was heated to 160 °C under an Argon atmosphere with magnetic stirring for 30 min until the solution became clear. The reaction was allowed to cooled to 50 °C, and then 0.30 g ammonium fluoride and 0.20 g sodium hydroxide in 20 mL anhydrous methanol was slowly injected. The solution was slowly heated to 75 °C with the temperature maintained for 20 min, and then was further heated to 120 °C to completely evaporate the methanol and water. The reaction was subsequently quickly heated to 300 °C (in about 20 min) and maintained at this temperature for 60 min. After cooling to room temperature, the obtained UCNPs were precipitated and washed by addition of ethanol and acetone, and separated by centrifugation at 8000 rpm for 10 min, with the process repeated three times. The purified pellets were re-dispersed in hexanes (~10 mL) for the subsequent shell growth. For shell growth, the same protocol was used as previously reported, and the separation and washing process were the same to that used in preparing the core. The purified core/shell UCNPs were kept in hexane at room temperature for future use.

### 3.4. Synthesis of Ligand-Free Water Soluble UCNP

Ligand-free UCNPs were prepared according to a reported method [28,29]. A quantity of 10 mg of the as-synthesized UCNPs was dispersed in 2 mL acidic H_2_O (pH 4, by adding 0.1 M HCl) followed by stirring for about 3 h. The solution was then extracted at least three times using hexane to remove any soluble oleic acid. The ligand-free UCNPs in the water phase were collected and washed by centrifugation, and were finally re-dispersed in H_2_O.

### 3.5. Coating the Ligand-Free UCNP with Sodium Citrate

Sodium citrate coated UCNPs were prepared using a previously reported method [30]. Sodium citrate was added into a 1-mg·mL^−1^ ligand-free UCNP water solution with a final concentration of 5 mg·mL^−1^. Then, the solution was sonicated for 60 min, and the sodium citrate coated UCNPs (SC-UCNP) were ready to use.

### 3.6. Conjugation of Avidin onto SC-UCNP

To eliminate the free sodium citrate in the SC-UCNP solution, 0.3 mL of solution was centrifuged in a filter tube (cut-off size: 100 kDa) at 6000 rpm for 5 min, and then washed with 4-(2-hydroxyethyl) piperazine-1-ethanesulfonic acid buffer (HEPES, pH 7.2) twice. The SC-UCNPs were re-suspended in 300 μL HEPES buffer. A volume of 100 μL of “activation solution” containing 2 mg 1-Ethyl-3-(3-dimethylaminopropyl) carbodiimide (EDC) and 1 mg Sulfo-NHS (*N*-hydroxysulfosuccinimide) (Sulfo-NHS) freshly dissolved in 1 mL HEPES buffer) was added and the solution was stirred for 15 min. Then the solution was centrifuged again to separate the UCNPs from the free EDC/Sulfo-NHS. The activated SC-UCNPs were re-suspended in HEPES buffer for further conjugation. A volume of 50 μL avidin solution (5 mg·mL^−1^) was added into the UCNP solution followed by stirring at room temperature for 2 h. The solution was then filter centrifuged to separate the UCNPs from free avidin in solution, and the obtained UCNPs were washed twice using HEPES buffer. Finally, the avidin-UCNPs were re-dispersed in 0.3 mL borate buffer (50 mM, pH 9.25).

### 3.7. Immobilization of Avidin-UCNPs on Paper Substrates and Bioconjugation of Oligonucleotide Probe

Physical adsorption was used in order to immobilize avidin-UCNPs onto cellulose paper substrates as was demonstrated in our previous studies [16,17]. Briefly, a 2.0 μL aliquot of avidin-UCNPs solution at 1 mg·mL^−1^ concentration was spotted on each of the paper arrays (spot inner diameter *ca.* 3 mm) that were localized by pre-printing a hydrophobic wax ring onto the paper substrates using a Xerox ColorQube 8570 DN (Xerox Canada, Toronto, Canada) solid ink printer. The paper substrates were subsequently dried under vacuum after spotting. The paper substrates were then rinsed with borate buffer for about 10 min in order to wash off any loosely bound avidin-UCNPs and subsequently dried under vacuum. For the bioconjugation of biotinylated *E. coli* hlyA probe, a 2 μL aliquot of oligonucleotide probe solution at 10 μΜ concentration that was dissolved in borate buffer was spotted onto the paper arrays that was modified with immobilized avidin-UCNPs. The paper substrates were incubated for 1 h at room temperature and subsequently washed with borate buffer containing 100 mM NaCl.

### 3.8. DNA Hybridization Assay

Unless otherwise stated, all the hybridization assays were done using the oligonucleotide target solution that was dissolved in 50 mM borate buffer (pH 9.25, 100 mM NaCl). Prior to the hybridization assays, the surface of UCNPs was passivated for 30 min with a blocking solution that comprised 1% BSA and 0.05% Tween 20 in order to ameliorate non-specific adsorption of DNA onto the immobilized UCNPs. Hybridization assays were conducted by spotting a 2.0 μL aliquot of *E. coli* hlyA target solution at various concentrations onto the paper arrays for 5 min. Subsequently, a 2.0 μL aliquot of Cy3-labeled reporter DNA at 8.0 μM concentration was spotted onto the paper arrays and incubated for 5 min. The paper substrates were subsequently washed with borate buffer and dried under vacuum prior to image scanning using an epifluorescence microscope that was equipped with a NIR laser excitation source (980 nm, 800 mW).

### 3.9. Selectivity Experiments

For one base pair mismatch discrimination experiments, solutions of fully-complementary target (FCT), one base pair mismatch (1BPM) target and non-complementary (NC) target at equi-molar concentrations (4.0 μM) were individually spotted onto the paper arrays and incubated for 5 min at room temperature. Subsequently, Cy3-labeled reporter at 8.0-μM concentration was spotted and incubated for additional 5 min. The paper substrates were then washed and allowed to dry prior to data collection. To improve the stringency of hybridization, the paper substrates were subsequently exposed to borate buffer containing 20% (*v*/*v*) formamide for 30 min and subsequently scanned for data collection after they had been dried under vacuum. For evaluation of the selectivity of hybridization in different background sample matrices, the target DNA (*E. coli* hlyA FCT) at 0.5-μM concentration was fortified with either salmon sperm DNA at *ca.* 3.1-μM concentration, Poly T sequence at 3.5-μM concentration, bovine serum albumin (BSA) at 400-μM concentration or undiluted goat serum. Four replicate measurements (*n* = 4) were taken for each of the sample solutions unless otherwise stated.

### 3.10. Data Analysis

For *E. coli* hlyA target detection, images corresponding to the emission band associated with UCNP PL (green channel), and images corresponding to the emission band associated with Cy3 PL (Cy3 channel) were analyzed. The images were opened and processed using ImageJ software (version 1.48 g, National Institutes of Health, Maryland, USA). After background signal subtraction, the intensity of each fluorescent spot was manually determined. The LRET ratios (*R*_image_) used for quantification were calculated using Equation (1):

*R*_image_ = (EM_Cy3_/EM_UCNP green_)_DA_ − (EM_Cy3_/EM_UCNP green_)_D_ (1)

where EM_Cy3_ is the total PL intensity for the image taken using the Cy3 emission filter (Cy3 channel), while EM_UCNP_ is the PL intensity for the image taken using the green emission filter (UCNP channel). The subscript DA indicates a measurement conducted in the presence of both donor and acceptor, while D indicates the measurement made without acceptor (control).

## 4. Conclusions

LRET-based sandwich format hybridization assays have been developed for determination of unlabeled oligonucleotide target on cellulose paper using UCNPs as energy donors and Cy3-labeled oligonucleotide reporters as energy acceptors. The hybridization of Cy3-labeled reporter DNA and the unlabeled target that was pre-hybridized with surface-bound probe DNA provided the proximity between Cy3 and UCNP for the LRET process. A ratiometric method was optically stimulated by illuminating the UCNPs with a 980 nm laser. A LOD of 146.0 fmol was obtained by the sandwich format assay, with the LRET efficiency being attributed to the distance between the donor and acceptor in a sandwich format. Selectivity was tuned by use of formamide, and achieved a maximum signal ratio of 1.81 between FCT and 1BPM. The sandwich format hybridization assay remained functional even in undiluted serum, and this suggests potential for oligonucleotide determination in samples of clinical origin where the background matrix can be very complicated. Thus far, bench-top fluorescent microscope is used to collect the signal, but we expect that smartphone cameras could be a replacement, making this assay more portable and low-cost.
